# The influence of environmental conditions on kinetics of arsenite oxidation by manganese-oxides

**DOI:** 10.1186/s12932-015-0030-4

**Published:** 2015-09-16

**Authors:** Matthew H. H. Fischel, Jason S. Fischel, Brandon J. Lafferty, Donald L. Sparks

**Affiliations:** Department of Plant and Soil Sciences, Delaware Environmental Institute, University of Delaware, 221 Academy Street, 250A ISE Lab, Newark, DE 19711 USA; Engineer Research and Development Center, U.S. Army Corps of Engineers, 3909 Halls Ferry Rd., Vicksburg, MS 39180 USA

**Keywords:** Manganese-oxide, Arsenic, Kinetics, Redox, Biogenic manganese-oxides

## Abstract

**Background:**

Manganese-oxides are one of the most important minerals in soil due to their widespread distribution and high reactivity. Despite their invaluable role in cycling many redox sensitive elements, numerous unknowns remain about the reactivity of different manganese-oxide minerals under varying conditions in natural systems. By altering temperature, pH, and concentration of arsenite we were able to determine how manganese-oxide reactivity changes with simulated environmental conditions. The interaction between manganese-oxides and arsenic is particularly important because manganese can oxidize mobile and toxic arsenite into more easily sorbed and less toxic arsenate. This redox reaction is essential in understanding how to address the global issue of arsenic contamination in drinking water.

**Results:**

The reactivity of manganese-oxides in ascending order is random stacked birnessite, hexagonal birnessite, biogenic manganese-oxide, acid birnessite, and δ-MnO_2_. Increasing temperature raised the rate of oxidation. pH had a variable effect on the production of arsenate and mainly impacted the sorption of arsenate on δ-MnO_2_, which decreased with increasing pH. Acid birnessite oxidized the most arsenic at alkaline and acidic pHs, with decreased reactivity towards neutral pH. The δ-MnO_2_ showed a decline in reactivity with increasing arsenite concentration, while the acid birnessite had greater oxidation capacity under higher concentrations of arsenite. The batch reactions used in this study quantify the impact of environmental variances on different manganese-oxides’ reactivity and provide insight to their roles in governing chemical cycles in the Critical Zone.

**Conclusions:**

The reactivity of manganese-oxides investigated was closely linked to each mineral’s crystallinity, surface area, and presence of vacancy sites. δ-MnO_2_ and acid birnessite are thought to be synthetic representatives of naturally occurring biogenic manganese-oxides; however, the biogenic manganese-oxide exhibited a lag time in oxidation compared to these two minerals. Reactivity was clearly linked to temperature, which provides important information on how these minerals react in the subsurface environment. The pH affected oxidation rate, which is essential in understanding how manganese-oxides react differently in the environment and their potential role in remediating contaminated areas. Moreover, the contrasting oxidative capacity of seemingly similar manganese-oxides under varying arsenite concentrations reinforces the importance of each manganese-oxide mineral’s unique properties.

## Background

Manganese (Mn)-oxides are important minerals in the environment because of their abundance and high reactivity. They are among the strongest oxidants in soils and sediments, and generally have high sorption capacities. These characteristics make Mn-oxides’ role paramount in sequestering both naturally occurring and anthropogenic toxins in the environment. The structure, crystallinity, and particle size of Mn-oxides are important factors which determine reactivity and thus the relative rate of Mn oxidation in the subsurface environment.

Manganese-oxide minerals are composed of MnO_6_ octahedra, which can be arranged in numerous ways. Subtle variations in the arrangement of these octahedra give rise to a multitude of minerals with nearly the same crystalline structure. Many naturally occurring Mn-oxides are difficult to characterize because they are small and exhibit poorly crystalline structure. Generally, Mn-oxides fit into two broad structural groups: tunnel structures and layer structures. Layered structures are characterized by stacked sheets of Mn octahedra with cations or water molecules integrated into the region between octahedral layers. Tunnel structures differ by consisting of a sequence of edge-sharing Mn octahedra which link corners to make tunnels with a rectangular or square cross section. Manganese-oxides exhibiting layered structure tend to be more reactive than those with tunnel structure due to the impact of the mineral configuration on surface area, vacancy and edge sites, and Mn(III) substitution. Some variations of the layered structure exhibit a triclinic configuration, including random stacked birnessite. However, among Mn-oxides with layered structure, those with configurations similar to hexagonal birnessite tend to be the most reactive and important in the environment [1].

Acid birnessite, biogenic Mn-oxide, hexagonal birnessite, and hydrous δ-MnO_2_ are Mn-oxides that exhibit a similar hexagonal structure. Two kinds of reactive sites exist in birnessite type minerals with hexagonal structure: vacancy and edge sites [2, 3]. Vacancy sites are areas where Mn is missing within the Mn(IV) octahedral sheets, while edge sites are found on the edges of the octahedral sheets. Mn-oxides with this configuration derive some of their negative charge and reactivity from the presence of vacancy sites [4]. Differences in reactivity between minerals sharing the hexagonal birnessite structure can be partially attributed to variances in the amount of these vacancy and edge sites [4]. Based on scanning and transmission electron microscopy, hexagonal birnessite and acid birnessite have a more ordered crystalline structure compared to δ-MnO_2_ and biogenic Mn-oxide. A more ordered structure tends to indicate a lower surface area and fewer reactive vacancy and edge sites, leading to decreased reactivity. Accordingly, acid birnessite is reported to have a surface area of 36 m^2^ g^−1^ and 12.0 % vacancies, while δ-MnO_2_ was found to have a surface area of 121 m^2^ g^−1^ and 6.0 % vacancies [5]. The greatly increased surface area of δ-MnO_2_ leads to a high reactivity as also seen in the biogenic Mn-oxide with 16.7 % vacancies and similar amorphous structure [5]. While vacancy sites are important for sorption of many cations, the influence of edge sites on the reactivity of these Mn-oxides should not be overlooked [6]. Reaction products, such as Mn(II), can also fill vacancy sites, or sorb on edge sites, blocking sorption and reactions with other compounds. Accordingly, edge sites can lead to higher reactivity in a mineral like acid birnessite, even though it has a greater proportion of total vacancy sites. In addition to structure, origin can greatly influence Mn reactivity. Many of the Mn-oxide minerals found in the environment are created by soil microorganisms and exhibit a layered structure similar to hexagonal birnessite [3, 4, 6].

Even over a large range of pHs, Mn-oxides play an important role in oxidizing many redox sensitive metals in the environment [7]. For example, they are one of the main agents that oxidize toxic and mobile arsenite [As(III)], to arsenate [As(V)], which is less mobile and less toxic [8]. After this reaction, As(V) can be readily removed through precipitation or adsorption to minerals such as Mn-oxides and Fe-oxides [9]. As the reaction between As(III) and Mn-oxides continues, the surface of some Mn-oxides may become passivated, resulting in a biphasic kinetic reaction [9–11]. Initially, the reaction proceeds rapidly; however, as the As(III) oxidation continues, intermediates including Mn(II) and Mn(III) along with As(V) may sorb to the Mn-oxide surface or replace surficial Mn(IV), which leads to a decrease in the oxidation rate during the later phase of the reaction [10].

The toxic impacts of As contamination are a global issue that affects the health and wellbeing of many diverse populations. It is estimated that over 150 million people worldwide are endangered by As contamination [12]. Arsenite has the greatest impact on human health in a few critical developing countries where natural geological formations contain hazardous levels of As. The World Health Organization deemed Bangladesh “the largest mass poisoning of a population in history,” due to the installation of wells in naturally As contaminated areas [13, 14]. These shallow wells often tap into alluvial sediments that are nearly devoid of oxidizing agents such as Mn and Fe and are rich in As(III) accumulated from weathering of upstream rocks. The pH of these soils also impacts As transport, with older deposits ranging from pH 6.0–6.5 and more recent alluvium having a pH of 7.0–8.5 [15].

Understanding the structure of different Mn-oxides can provide critical insight to their predicted reactivity. However, it is still unclear how many of the Mn-oxides behave under changing environmental conditions. Very few studies have examined the effects of variations in concentration of reactants and temperature on Mn-oxides reactivity. Even pH studies have only encompassed a limited selection of the many Mn-oxides. This study seeks to provide critical information on the reactivities of several Mn-oxides in oxidizing As(III) under varying pH, temperature, and As(III) concentration. Such information will provide important insights on fate and transport of toxic As(III) in the soil environment.

## Results and discussion

### Effect of Mn-oxide structure on As(III) oxidation kinetics

**Fig. 1:**
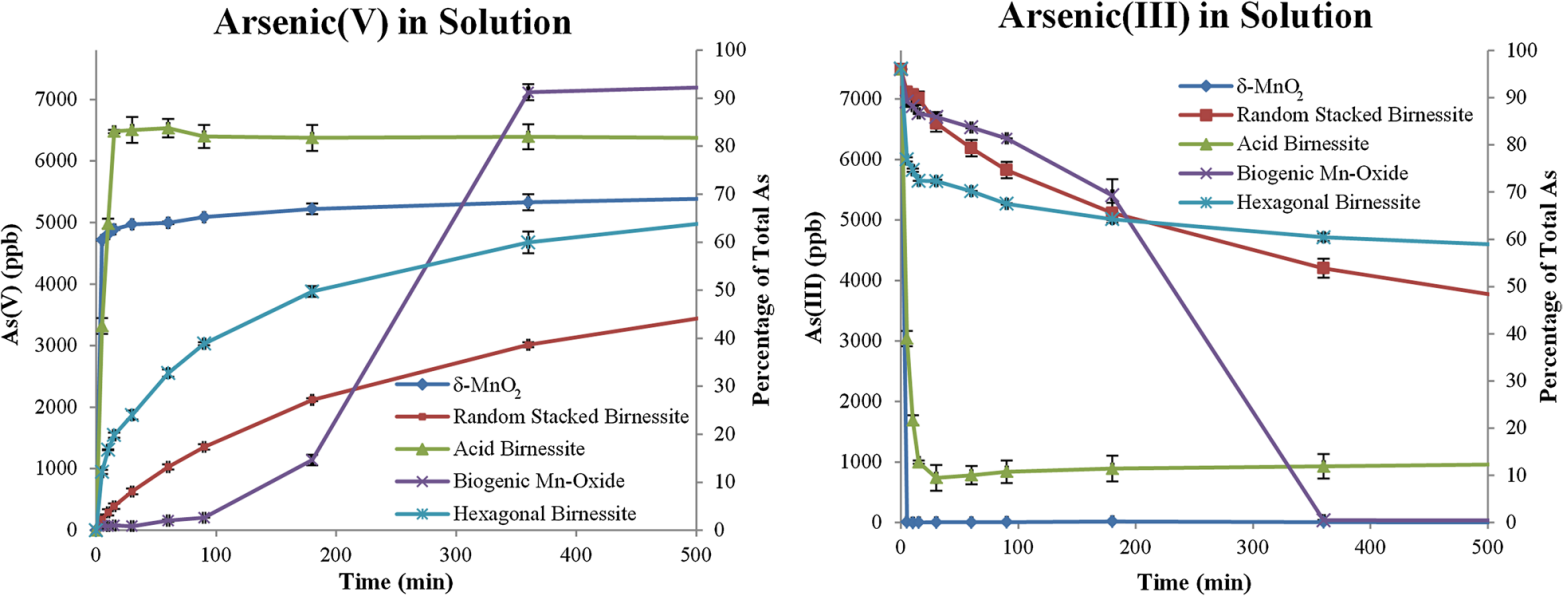
Oxidation of arsenite by manganese-oxides: the oxidation of arsenite by five manganese-oxides at 1.82 mM Mn, 100 μM As(III), and pH 7.2. The formation of arsenate (*left*) from the oxidation of arsenite (*right*) for the first 500 min of the reaction

Based on structural assumptions, the predicted reactivity of the Mn-oxides investigated in this study in descending order should be δ-MnO_2_ ~ biogenic Mn-oxide > acid birnessite > hexagonal birnessite > random stacked birnessite. However, this is solely based on structure (i.e. particle size, surface area, and percentage of vacancy sites) and does not encompass the full scope of factors contributing to Mn-oxide reactivity. Batch reactions with 100 μM As(III) and 1.82 mM Mn-oxide at pH 7.2 are shown in Fig. 1. These data confirm that δ-MnO_2_ was the most reactive of the Mn-oxides studied and it oxidized ~100 % of the As(III) in the first 5 min. The δ-MnO_2_ sorbed on average ~30 % of the produced As(V) over the course of the experiment. In the first 4 h, acid birnessite was the second most reactive, followed by hexagonal birnessite, and finally biogenic Mn-oxide. Between 3 and 6 h the reactivity of biogenic Mn-oxide greatly increased and most of the As(III) in solution was oxidized. This initial delay in reactivity for the biogenic Mn-oxide was unexpected and may be attributed to the presence of remnant bacterial cell fragments interfering with the reactive sites on the Mn-oxide, as was found for iron oxides by Franzblau et al. [16]. Additionally, extracellular polymeric substances (EPS) may be present, further delaying the initial As(III) oxidation [17]. Remnant bacterial cells and EPS may also make the biogenic Mn-oxide more thermodynamically stable and cause increased persistence of the mineral phase. After this preliminary period of reacting slowly, the biogenic Mn-oxide proceeded to oxidize ~99 % of the As(III), releasing more oxidized As into solution than any of the other Mn-oxides studied. The δ-MnO_2_ reaction stopped in <5 min, while with acid birnessite the reaction finished in <15 min and oxidized 86 % of the total As in solution. All reactions containing Mn-oxides with hexagonal structure were complete after 1 day, except for hexagonal birnessite.

The rate constants of As(III) oxidation on the Mn-oxides were determined by fitting data to different kinetic models. All Mn-oxides tested fit well to a first-order kinetic model as demonstrated in Table 1. However, the δ-MnO_2_ reaction was completed in <5 min and the data were fit to a first-order model. The observed rate coefficients for the Mn-oxides show that δ-MnO_2_ oxidized As(III) about ten times more quickly than the next most reactive mineral, acid birnessite. The three remaining Mn-oxides were much less reactive as demonstrated by their significantly smaller k_observed_. However, from 90 to 180 min the biogenic Mn-oxide completely oxidized most of the As(III) solution. Due to the temporal distance between these two sample times it is probable that the oxidation occurred in much <90 min and that biogenic Mn-oxide is more reactive in a small time window than indicated by the reported k_observed_. Our results agree with the scientific literature in that reactions and back reactions of Mn and As have previously been linked to pseudo-first or pseudo-second-order kinetics [18–20].

**Table 1:** The data from fitting the initial five manganese-oxides’ oxidation reaction to first-order kinetic models

Manganese oxide	Time scale (min)	k_observed_ (s^−1^)	r^2^
Acid birnessite	0–15	0.1326	0.9829
δ-MnO_2_	0–5	1.3684	1.0000
Hexagonal birnessite	0–5	0.0446	1.0000
	5–2880	0.0003	0.8465
Biogenic Mn-oxide	0–30	0.0039	0.8229
	90–180	0.0212	0.9154
Random stacked birnessite	0–720	0.0012	0.9561

Only δ-MnO_2_ and biogenic Mn-oxide appeared to sorb a noticeable amount of As(V) during the oxidation reaction. This was determined by the total As(III) and As(V) in solution compared to the total concentration of As added initially. The δ-MnO_2_ initially sorbed approximately 37 % of the total As, which steadily decreased to 21 % over the course of the 2 days experiment. The biogenic Mn-oxide initially showed 5 % sorption after 5 min. During the highest rate of reaction at 3 h, approximately 13 % of the total As was sorbed. A larger number of edge sites in δ-MnO_2_ may be responsible for sorbing As(V) and thus removing it from solution [2].

Random stacked birnessite was the only Mn-oxide with triclinic structure, which has been shown to have decreased reactivity compared to layered Mn-oxides with hexagonal structure [21]. Triclinic birnessite is different from hexagonal birnessite in that the triclinic form has a minute lengthening of one of the unit cell planes, most likely resulting from an increase in Mn(III) [22–24]. Random stacked birnessite showed the slowest overall As(III) oxidation kinetics of all minerals studied and also has fewer edge sites compared to the minerals with hexagonal structure. The random stacked birnessite reaction fit best to a first-order kinetic model as seen in Table 1. The production of As(V) was still occurring at a slow rate when the experiment ended after 2 days. Additionally, the amount of total As [As(V) and As(III) combined] in solution was always equal to the initial concentration, indicating there was little to no sorption of the As(V) produced during the experiments.

### Effect of pH on manganese reactivity and arsenic(III) oxidation

**Fig. 2:**
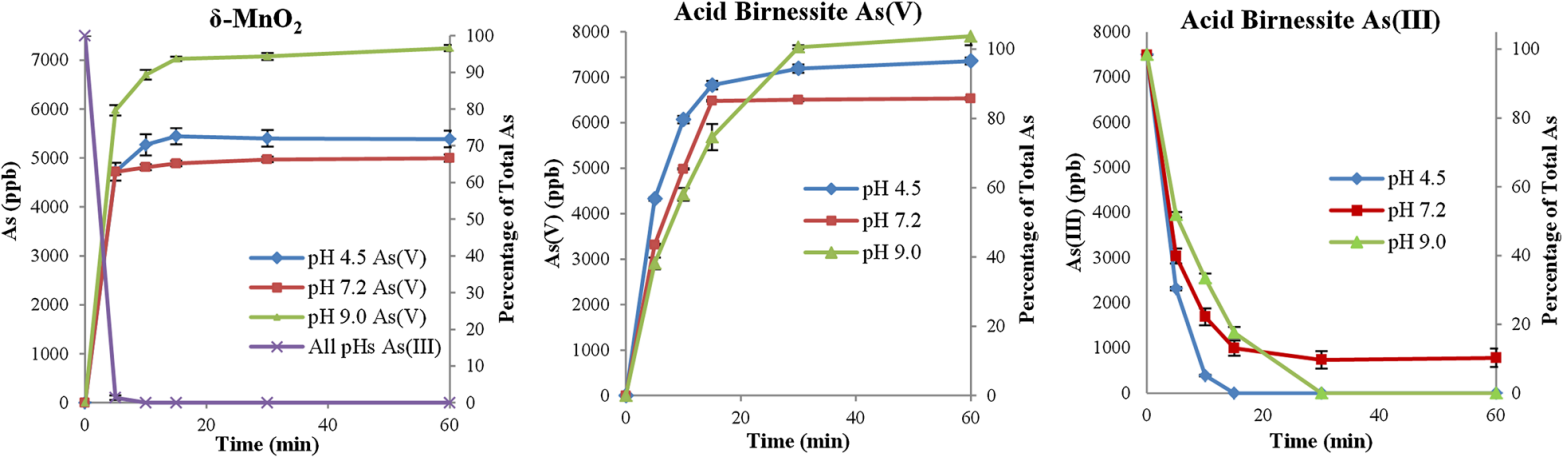
Effect of pH on manganese As(III) oxidation: the influence of pH on the oxidation of 100 μM arsenite by 1.82 mM Mn δ-manganese-oxide (*left*) and acid birnessite (*middle* and *right*). The minerals were reacted with As(III) at pH 4.5, 7.2, and 9.0. The oxidation of As(III) by the manganese-oxide produces As(V)

The pH of an environment plays a critical role in the reaction of Mn-oxides with As due to the importance of H^+^ in reactions governing the oxidation of As. Two of the most reactive Mn-oxides, δ-MnO_2_ and acid birnessite, were reacted with 100 μM As(III) at pH 4.5, 7.2, and 9.0 (Fig. 2). The influence of pH on As(III) oxidation of Mn-oxides is described by Feng et al. [25] in the following reactions. In acidic pH conditions, the reaction is represented as:

1 $$\left( {{\text{MnO}}_{2} } \right)_{x} + {\text{H}}_{3} {\text{AsO}}_{3} + 0.5{\text{H}}^{ + } = 0.5{\text{H}}_{2} {\text{AsO}}_{4}^{ - } + 0.5{\text{HAsO}}_{4}^{2 - } + {\text{Mn}}^{2 + } + \left( {{\text{MnO}}_{2} } \right)_{x - 1} + {\text{H}}_{2} {\text{O}} .$$

In this reaction, an H^+^ atom is consumed during the oxidation of As. In alkaline pH conditions, the result is the production of an H^+^ atom as seen in the reaction below:

2 $$\left( {{\text{MnO}}_{ 2} } \right)_{x} + {\text{H}}_{ 3} {\text{AsO}}_{ 3} = 0. 5 {\text{H}}_{ 2} {\text{AsO}}_{ 4}^{ - } + 0. 5 {\text{HAsO}}_{ 4}^{ 2- } + 1. 5 {\text{H}}^{ + } + \left( {{\text{MnO}}_{ 2} } \right)_{x - 1} \cdot {\text{MnO}} .$$

Thus, at a more alkaline pH this reaction will proceed quickly toward the products in order to produce more H^+^ ions in an effort to reach equilibrium. The species of As present also changes within the pH range. Based on the p*K*a of As species, at pH 4.5 As(III) is present as H_3_AsO_3_° and As(V) as H_2_AsO_4_^−^. At pH 7.2 the As(III) species is the same; however, As(V) is a nearly equal mixture of H_2_AsO_4_^−^ and HAsO_3_^2−^. When the pH is increased to 9.0, As(III) exists as approximately 40 % H_2_AsO_3_^−^ and 60 % H_3_AsO_3_°, while As(V) is completely HAsO_3_^2−^. Manganese-oxides also experience changes in chemical properties with pH alterations. As pH increases, the surface charge of Mn-oxides becomes increasingly negative. The point of zero charge for δ-MnO_2_ and acid birnessite occurs at pH 2.8 and 3.14, respectively, indicating the zeta potential of these minerals becomes increasingly negative as pH increases from 4.5 to 9.0 [1, 26]. These charge characteristics tend to promote oxidation of As(III) due to enhanced favorability of desorption of oxidized As(V) from reactive sites back into the solution [25]. However, this increased negative surface charge may result in more sorption of Mn(II) intermediates on the Mn-oxide surface, resulting in surface passivation and reduced As(III) oxidation.

The δ-MnO_2_ data in this study follow some of these assumptions as demonstrated in Fig. 2. The δ-MnO_2_ oxidized ~99 % of the As(III) in the first 5 min under all pHs. However, pH dependent differences were seen in the amount of sorbed As(V). The δ-MnO_2_ at pH 4.5 and pH 7.2 sorbed ~ 37 % of the As(V) before 5 min, while the δ-MnO_2_ at pH 9.0 sorbed ~20 % of the As(V) within 5 min. δ-MnO_2_ at pH 4.5 and 7.2 initially sorbed similar amounts of As(V); however, after 10 min the reaction at pH 4.5 had released ~9 % more As(V) into solution than the pH 7.2. The hypothesis that less sorption of As(V) would occur at higher pH was confirmed by the amount of total As in solution over the range of pHs in the δ-MnO_2_ experiments. The pH 4.5, 7.2, and 9.0 reactions had ~26, ~21, and ~6 % sorption of As(V), respectively, after 2 days. This was presumably caused by the increase in Mn(II) adsorption at higher pH, which Stone and Ulrich [27] also found.

**Fig. 3:**
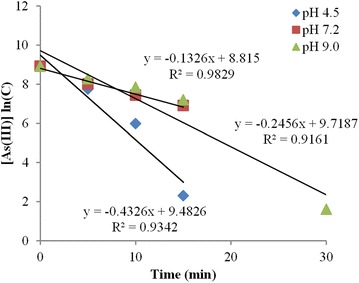
Acid birnessite first-order kinetic model: linear regression analysis of acid birnessite batch reactions at pHs 4.5, 7.2, and 9.0 with 100 μ mM As(III). The first-order kinetic model ln(C) versus time provides a best fit for the reactions

The acid birnessite oxidized As(III) differently over the range of pH investigated. Within the first 5 min, the acid birnessite at pH 4.5 produced the most As(V), followed by the pH 7.2, and pH 9.0. However, between 15 and 30 min the reaction at pH 9.0 continued to increase and equaled the amount of As(V) produced by acid birnessite at pH 4.5. Despite the initially slow reaction at pH 9.0, the data mimic the typical trend in oxidation kinetics for acid birnessite. The lower overall reactivity of acid birnessite at pH 7.2 is expected because most Mn-oxides experience a dip in reactivity somewhere in the pH range. This point of lowest reactivity is related to the redox half reactions (H_3_AsO_4_ + H^+^) and (MnO_2_ + H^+^)/Mn^2+^ in acidic conditions with redox potentials of 0.56 and 1.23 V, respectively; and under alkaline conditions MnO_2_/(Mn(OH)_2_ + OH^−^) and AsO_4_^3−^/(AsO_2_^−^ + OH^−^) with standard potentials of 0.1 and −0.71 V, respectively [25]. The pH where the lowest potentials of these two reactions is met determines the point where the Mn-oxides produce the least As(V). Additionally, in the acid birnessite reaction at pH 7.2 the As(III) was never completely oxidized and after 15 min–2 days ~ 1 ppm As(III) remained in solution. Unlike δ-MnO_2_, acid birnessite did not show significant As(V) sorption, and thus it reacted following the previous assumptions, with the reactions at pH 4.5 and 9.0 oxidizing all As(III) in solution to As(V). The data from each reaction fit well to a first-order kinetic model with r^2^ values >0.91 (Table 2). The observed rate coefficients for pH 4.5, 7.2, and 9.0 were 0.4326, 0.1326, and 0.2456 s^−1^, respectively, as demonstrated by the linear fitting of the natural log of As(III) concentration in Fig. 3. These data reinforce the idea of lowered Mn-oxide reactivity over the middle range of pH. The reaction was initially slower at pH 9.0, than pH 4.5, but eventually both reactions completely oxidized the As(III) in solution.

**Table 2:** The data from the first-order kinetic models of δ-MnO_2_ and acid birnessite with varying temperature, pH, and As(III) concentration

Experimental conditions	Time scale (min)	k_observed_ (s^−1^)	r^2^
δ-MnO_2_			
10 °C	5–90	0.0026	0.8574
25 °C	5–90	0.0027	0.9409
40 °C	5–90	0.0037	0.9414
100 μM As(III)	0–5	1.3684	1.0000
1 mM As(III)	5–90	0.00234	0.9271
10 mM As(III)	5–60	0.0011	0.8422
Acid birnessite			
pH 4.2	0–15	0.4326	0.9342
pH 7.2	0–15	0.1326	0.9829
pH 9.0	0–30	0.2456	0.9196
10 °C	0–90	0.0043	0.8972
25 °C	0–30	0.0131	0.8688
40 °C	0–10	0.0441	0.8532
100 μM As(III)	0–15	0.1326	0.9829
1 mM As(III)	0–30	0.0131	0.8688
10 mM As(III)	0–30	0.0143	0.8507

### Effect of temperature on arsenic(III) oxidation kinetics on manganese-oxides

**Fig. 4:**
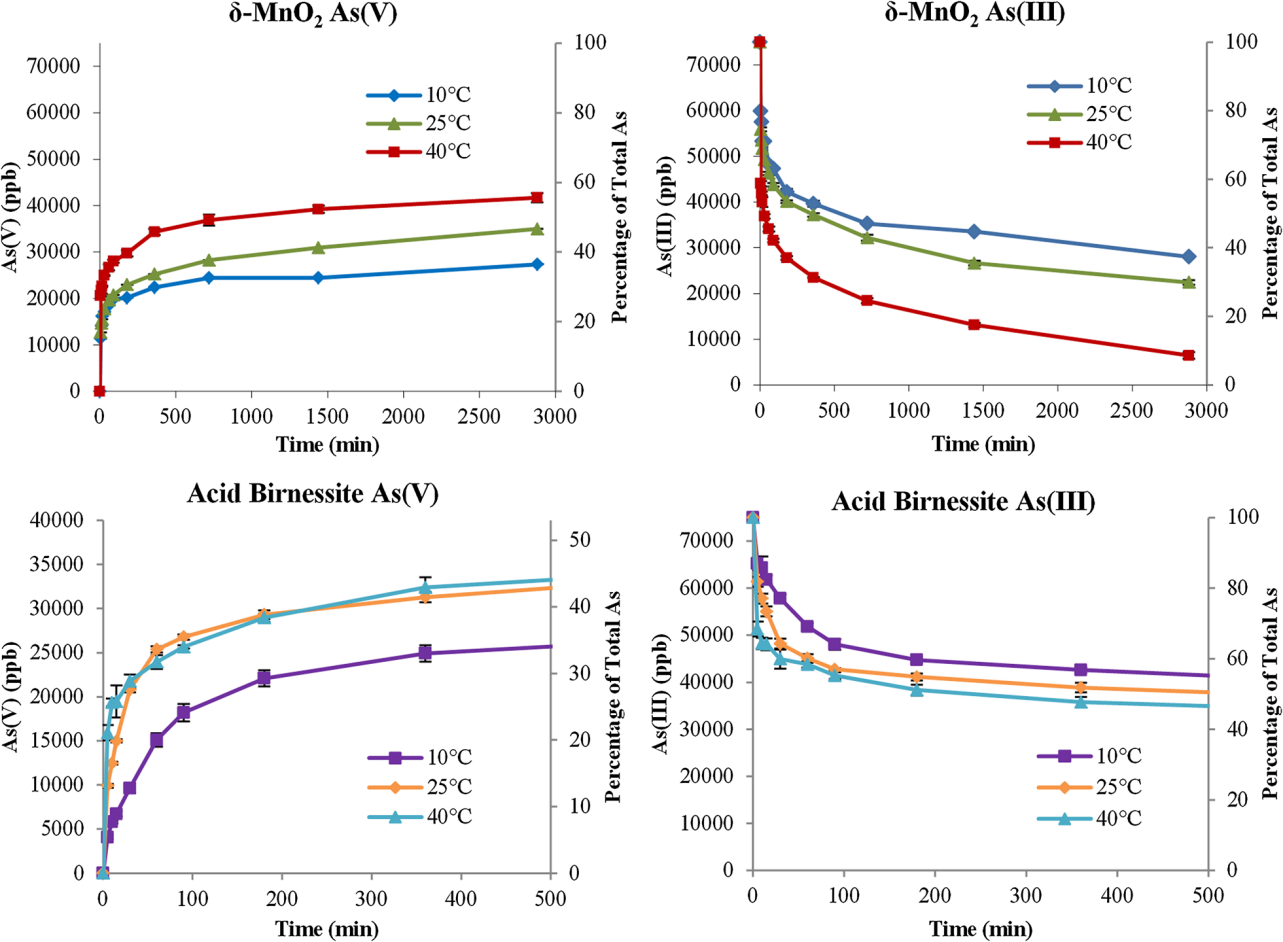
Effect of temperature on arsenic(III) oxidation by manganese-oxides: the influence of temperature on the oxidation of 1 mM arsenite by 1.82 mM δ-manganese-oxide (*top*) and acid birnessite (*bottom*). The minerals were reacted at 10, 25, and 40 °C at pH 7.2. The oxidation of As(III) (*right*) by a manganese-oxide produces As(V) (*left*)

The kinetics of As(III) oxidation by δ-MnO_2_ and acid birnessite relative to temperature were determined by reacting the minerals with 1 mM As(III) at 10, 25, and 40 °C as seen in Fig. 4. The As(III) oxidation by δ-MnO_2_, with respect to varying temperature, appeared to be intuitively linked, with increasing temperature resulting in a faster oxidation of As(III). Additionally, a ~10 % increase in As(V) sorption by δ-MnO_2_ was seen by increasing the temperature from 10 or 25–40 °C. The 10, 25, and 40 °C reactions with 1 mM As all fit first-order reaction kinetics (Table 2). With the k_observed_ = 0.0026, 0.0027, and 0.0037 s^−1^, respectively. The fast step of this reaction occurs within the first 5 min, making it challenging to define with a limited temporal scale. Thus, the first 5 min were excluded for these calculations and the reported coefficients represent the second and slower step of the reaction [11]. As discussed previously, there are several reactions occurring during the oxidation of As(III) by δ-MnO_2_ and it truly fits a pseudo first-order kinetic model.

The acid birnessite initially followed a similar trend, with increasing temperature the rate of reaction also increased. However, after 30 min the 25 and 40 °C reactions had produced the same amount of As(V). This may be due to the 40 °C reaction quickly proceeding with the first and fast step of As(III) oxidation and then slowly oxidizing the remaining As(III). All of the acid birnessite data fit well to a first-order kinetic model. The k_observed_ for the 10, 25, and 40 °C acid birnessite reactions based on the first-order model are 0.0043, 0.0131, and 0.0441 s^−1^, respectively (Table 2). The increase in reactivity with a higher temperature may be partially explained by the fact that with a rise in temperature the point of zero charge of the Mn-oxide’s surface decreases [28, 29]. This causes the surface of the Mn-oxide to become more negatively charged and in turn promotes the oxidation of As(III).

### Effect of As(III) concentration on oxidation kinetics

**Fig. 5:**
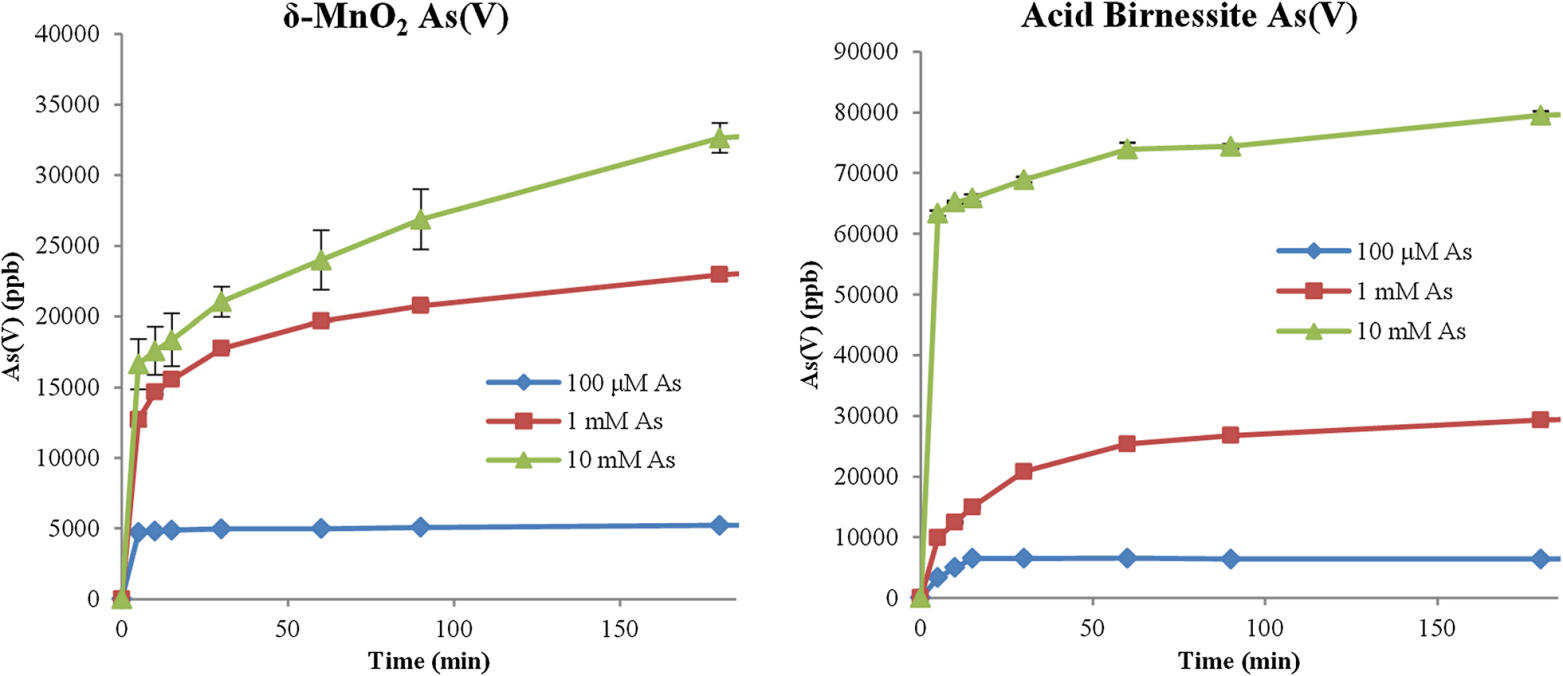
Effect of As(III) concentration on manganese oxidation kinetics: the oxidation of arsenite by 1.82 mM δ-manganese-oxide (*left*) and acid birnessite (*right*). Concentrations of initial As(III) reacted include 100 μM, 1, and 10 mM at pH 7.2

Acid birnessite and δ-MnO_2_ were reacted with 100 μM, 1, and 10 mM As(III) to determine the minerals’ oxidative capacity and the rate of reaction under varying As concentrations (Fig. 5). With increasing As(III) concentration in solution the two Mn-oxides reacted somewhat differently. The δ-MnO_2_ was much more sensitive to increases in As(III) than the acid birnessite, but both minerals were able to oxidize less of the total As(III) in solution with increasing As(III) concentration. It is possible that there is much more surface passivation on δ-MnO_2_ with the byproducts Mn(II), Mn(III), and perhaps As(V) during the course of the As(III) oxidation reaction compared to acid birnessite [10]. At 100 μM and 1 mM As(III) concentrations, oxidation by δ-MnO_2_ initially produces more As(V) in solution than acid birnessite, but after 10 and 15 min, respectively, acid birnessite releases more As(V) into solution. After 30 min at 1 mM As(III), the acid birnessite generated more As(V) than δ-MnO_2_. At 10 mM As(III) acid birnessite produced much more As(V) than δ-MnO_2_ for the entire course of the reaction. The greatest difference in the amount of 10 mM As(III) oxidized is reached at 30 min when the δ-MnO_2_ and acid birnessite have reacted with ~8 and ~19 % of the As(III), respectively. Acid birnessite oxidation of 100 μM, 1, and 10 mM As(III) fit first-order kinetic models with k_observed_ of 0.1326, 0.0131, and 0.0143 s^−1^ respectively (Table 2). With increasing As(III) concentration the rate coefficient of the acid birnessite reaction rose from 100 μM to 1 mM As(III) and then stayed somewhat constant between 1 and 10 mM As(III) indicating the reaction may proceed faster with greater As(III) concentration up to a limit. The δ-MnO_2_ fast reaction reached near completion in <5 min for the 100 μM As(III) reaction, which was fitted to a first-order kinetic model with k_observed_ equal to 1.3684. The 1 mM and 10 mM As(III) reactions fit first-kinetic models from 5–90 to 5–60 min, respectively, with k_observed_ equal to 0.00234 and 0.0011 s^−1^, sequentially. The δ-MnO_2_, at 1.82 mM, may have reached its upper limit in oxidation between 10 and 1 mM As(III), as little increase in As(V) production was observed with a tenfold increase in As(III).

## Experimental

### Manganese-oxide synthesis

The δ-MnO_2_ was chosen for this study due to its high reactivity and structural similarity to biogenic Mn-oxides [4]. The low degree of stacking in the layered structure of δ-MnO_2_ is the signature of this type of hexagonal birnessite [30–34]. Synthesis procedures followed an altered method of Murray found in Zhu [34]. 250 mL of 0.15 M Mn(NO_3_)_2_·4H_2_O were added at 20 mL min^−1^ into a 250 mL solution of 0.1 M KMnO_4_ in 0.2 M NaOH [34]. The reaction was mixed well during the addition of Mn(NO_3_)_2_. The Mn solid was obtained through centrifugation and then washed with DI water to ensure purity. Only fresh δ-MnO_2_ was used.

Acid birnessite was synthesized by following the procedures in Zhu [34]. First, a concentrated solution of HCl totaling 65.4 mL was added at 1 mL per min into a 1 L 0.4 M solution of boiling KMnO_−4_. The reaction was stirred well during the addition of acid. The Mn solid was collected through centrifugation and washed with DI water several times, until the remaining KMnO_4_ was removed as indicated by a clear supernatant.

Hexagonal birnessite was synthesized through a modified procedure described by Zhu [34]. 1 g of random stacked birnessite was added to 100 mL of 0.1 M NaNO_3_. The pH of the solution was adjusted to 5.0 through the addition of 0.5 M HNO_3_^−^. The pH was held constant through the use of a Metrohm pH stat. After 36 h of equilibrium, the Mn solid was centrifuged and washed several times to ensure purity.

Synthesis of random stacked birnessite was carried out using the procedure of Zhu [34], which was modified to exclude post-hydrothermal aging. Initially, 250 mL of 8 M NaOH and 250 mL of 0.4 M MnCl_2_ solutions were created using anoxic DI water and nitrogen gas was used to purge the solutions. The two solutions were cooled in a water bath to 0 °C before and during their mixing. The mixture of the two solutions was purged with nitrogen gas for the first half hour of the reaction, then a white pyrochroite [Mn(OH)_2_] suspension formed. After half an hour, the solution was vigorously mixed and aerated with oxygen gas for 5 h to oxidize the pyrochroite into random stacked birnessite, denoted by the formation of a black suspension. The suspension was then washed several times with DI water and stored.

Biogenic Mn-oxide was synthesized following the procedure of Zhu [34]. *Pseudomonas putida* strain GB-1 was cultured in 500 mL of *Leptothrix discophora* media in 2 L Erlenmeyer flasks at 30 °C and agitated at 200 rpm in a thermostatic shaker. Bacteria inoculums were created by culturing *P. putida* in media containing trace elements, mineral salts, and glucose for 12 h at 30 °C. After 36 h of cultivation, the Biogenic Mn-oxide was collected and centrifuged at 12,000 rpm, decanted and washed with DI water multiple times.

### Batch reactions

The reactivity of Mn-oxides for As(III) was investigated with a series of batch reactions. All experiments were run with a background electrolyte of 10 mM NaCl, and unless otherwise indicated, were buffered to pH 7.2 with 5 mM 3-(*N*-morpholino)propanesulfonic acid (MOPS). The background electrolyte, MOPS buffer, Mn-oxide solution, and DI water were added to a volume of 250 mL in a 500 mL flask. All Mn-oxides were used at a final concentration of 1.82 mM. The experiments were started with the addition of As(III). The flasks were shaken at 120 rpm to facilitate constant mixing of the solution. Samples were taken in 4.5 mL aliquots and filtered with 0.20 μm sterile syringe filters upon collection. After the start of the reaction, samples were collected at 5, 10, 30 min, 1, 1.5, 3, 6, 12, 24, and up to 48 h. All reactions were simultaneously run in triplicate to ensure reproducibility.

Initially, acid birnessite, hexagonal birnessite, random stacked birnessite, δ-MnO_2_, and biogenic Mn-oxide were reacted under the parameters described above, with the addition of 100 μM As(III). The two most reactive Mn-oxides, acid birnessite and δ-MnO_2_, were selected for further experimentation. δ-MnO_2_ and acid birnessite reactions with As(III) concentrations of 1 and 10 mM, were also investigated. A series of temperature controlled experiments with these two Mn-oxides were completed utilizing a temperature controlled chamber under 10, 25, and 40 °C with 1 mM As(III). Further experimentation controlling pH of the reaction at 25 °C was done with a Metrohm pH stat titrator at pH of 4.5, 7.2, and 9.0 with 100 μM As(III). The As(III) data from each experiment were fit to zero-order, first-order, and second-order kinetic models and the model with the best fit was chosen to represent the reaction as shown in Fig. 3.

### As analysis

The collected As(V) and As(III) from the batch reactions were filtered with 0.20 μm sterile syringe filters, diluted to an acceptable concentration, and then analyzed with liquid chromatography inductively coupled plasma mass spectrometry (LC-ICP-MS) for As(III) and As(V) in solution.

## Conclusions

All five Mn-oxides were capable of oxidizing a substantial amount of As(III) into As(V) within 2 days and Mn-oxide reactivity was closely related to structure and vacancy site differences. Experiments with δ-MnO_2_ and acid birnessite showed the importance of pH, temperature, and As(III) concentration in affecting the kinetics of As(III) oxidation. These results have critical implications for understanding and predicting the reactivity of Mn-oxides and mobility of As in the environment.

The effect of temperature on the kinetics of As(III) oxidation is especially relevant and important for soil systems because of the temperature difference between the ambient environment and subsurface. The long term reactivity of Mn-oxides should be much lower in soil than under many experimental conditions because of decreased temperatures in soil relative to most laboratory experiments. A primary environmental concern of As is contamination in groundwater and understanding the kinetics of As oxidation under these conditions is essential to fully comprehend the potential for this contamination under different subsurface conditions. Groundwater temperature is innately linked to surface temperature, and thus the climate of the region will have a significant impact on the subsurface reactivity of Mn-oxides. In the tropics, Mn-oxides will be more reactive because the underground temperature is close to 25 °C, while the same reaction will proceed more slowly in the 10 °C conditions found along the Mid-Atlantic United States and in other temperate regions. Similarly, As fate and mobility in the environment can be predicted based on soil pH. In Bangladesh, the older alluvial sediments have a lower pH than the more recent deposits. Knowing As sorption on Mn-oxides is greater in acidic conditions than in alkaline environments indicates the older sediments will sorb more As from the contaminated groundwater. Wells can then be drilled strategically into regions with older sediment deposits to mediate the risk of arsenic imbibition. As discussed previously, Mn minerals experience a dip in reactivity under certain pHs and reactivity can be enhanced by adjusting pH. Remediation strategies for sites contaminated with redox sensitive metals can benefit from the results of this study by realizing that the oxidation capacity of Mn-oxides can be bolstered by adding sulfur or lime amendments to adjust soil pH. Finally, varying the concentration of As(III) showed that concentration can greatly impact a Mn-oxide’s ability to react. Some Mn-oxides have greater total oxidation capacities and can produce more As(V) under higher concentrations of As(III), such as acid birnessite. While other Mn-oxides can react much more rapidly and oxidize nearly all As(III) into As(V) in short times at lower Mn to As(III) concentrations. Manganese-oxides are one of the most important minerals in the soil environment with respect to reactivity. This study provides insight into their chemical behavior under varying environmental conditions and its impacts on redox sensitive compounds.
